# Effect of internal phase particle size on properties of site mixed emulsion explosive at plateau environment

**DOI:** 10.1038/s41598-024-59215-z

**Published:** 2024-04-12

**Authors:** S. D. Xie, X. Y. Cai, H. B. Wu, Q. Wang, Z. R. Guo, Z. Y. Chen, C. S. Ma

**Affiliations:** 1 School of Architecture and Construction, Anhui University of Science & Technology, Huainan, 232001 China; 2 School of Chemical and Blasting Engineering, Anhui University of Science & Technology, Huainan, 232001 China

**Keywords:** Site mixed emulsion explosive, Plateau environment, Detonation velocity, Shock wave pressure, TG-DTG, Chemical engineering, Civil engineering

## Abstract

To study the effect of internal particle size on the microstructure properties and thermal decomposition characteristics of site mixed emulsion explosive at different altitudes. Site mixed emulsion explosive was prepared with different shear rate. The particle size, viscosity, sensitized bubbles, detonation velocity and peak pressure of the emulsion explosive were tested after stored at different simulated altitudes. The thermal decomposition characteristics of emulsion matrix prepared at three different rotational speeds were measured by thermogravimetric analyzer and kinetic analysis was performed by non-isothermal model Kissinger–Akah–Sunose (KAS) method. The results show that with the increase in altitude, the internal phase size showed a trend of first increasing and then decreasing, and the number of sensitized bubbles within the emulsion explosive decreases. At an altitude of 0 m, the detonation velocity and peak overpressure of the emulsion explosive prepared by 1600 r min^−1^ increased 4.78% and 29.09%, respectively compared with 1200 r min^−1^, and at an altitude of 4500 m, the detonation velocity increased 11.87%, the peak overpressure increased 43.98%. The thermal decomposition activation energy of the emulsion matrix at 1600 r min^−1^ increased 13.14% compared to 1200 r min^−1^. It shows that in the production of site mixed emulsion explosive at high altitude, reducing the particle size of the internal phase of emulsion explosives in a certain range can effectively improve the performance of emulsion explosives.

## Introduction

Emulsion explosive is one of the most widely used industrial explosives^1^. Site mixed emulsion explosive has become a popular development direction in the civil blasting industry due to its outstanding advantages such as high production efficiency, safety, convenience and low cost. In high-altitude areas such as Xinjiang and Inner Mongolia, which are rich in mineral resources in China. When the common site mixed emulsion explosive was used for engineering blasting, the performance changed easily and resulting in semi-explosive and non-explosive situations, which seriously affected the blasting safety of the project and the construction progress. Moreover, different altitudes also have different effects on site mixed emulsion explosive. Therefore, the improvement of the production process and formula of site mixed emulsion explosive in plateau environment has received extensive attention^2^. By simulating the plateau environment, Wu et al.^3^ studied the influence of temperature and air pressure changes on emulsion explosives and found that the sensitized bubbles of chemically sensitized emulsion explosives began to expand and accumulate and gradually become invalid bubbles at an altitude above 2500m. Gao et al.^4^ studied the effect of high altitude pressure on explosion performance and found that the explosion performance of expanded ammonium nitrate explosive was almost unaffected by high altitude pressure. Wu et al.^5^ used TG technology to analyze the influence of different internal phase particle sizes on the thermal decomposition characteristics of emulsion explosives. Yan et al.^6^ believed that under the same formula and process, the smaller the internal phase particles of emulsion explosive, the narrower the particle size distribution and the better the thermal stability, which was conducive to the safe production and long-term storage of explosive. Sun et al.^7^ analyzed the thermal stability of emulsion matrix with waste oil by TG-DSC technology and found that the thermal stability of the matrix and the critical temperature of thermal explosion could be improved by increasing the proportion of emulsifier. Liu et al.^8^ analyzed the explosive performance of emulsion explosive by changing the content of sensitizer and the particle size of internal phase, and found that the explosion effect was better when the particle size of internal phase was within 5μm and the content of sensitizer was 0.3%. Reynolds et al.^9^ analyzed that the crystallization of emulsion matrix is mainly related to micelle concentration and interfacial strength. However, there are few reports on the effect of internal phase particle size on the performance of site mixed emulsion explosive in plateau area.

At present, there are serious problems in the use and storage of mixed emulsion explosive in high altitude area. Due to the influence of low temperature and pressure, the explosive is easy to demulsification and crystallization and the number of effective bubbles is reduced, which resulting in the detonation performance can not meet the practical needs of engineering.

In this work, prepared site mixed emulsion explosives with different internal phase particle sizes by changing the shear rate, the differences between the microstructures of these emulsion explosives after storage in plateau environment were studied, and the explosive performance and thermal stability were analyzed. In order to provide guidance for the production of suitable plateau site mixed emulsion explosive.

## Experimental part

### Reagents

Experimental reagents: Ammonium Nitrate (AN) were obtained from Yongchang Nitro Fertilizer Co., LTD. (Henan, China); T154 Emulsifier were obtained from Xiangke Chemicals Co., Ltd. (Anqing, China). 0# diesel oil and engine oil were supplied Aladdin Reagent Co., LTD. (Shanghai, China), Citric acid and Sodium nitrite were obtained from Guoyao Chemicals Co., Ltd. (Shanghai, China).

### Preparation and storage environment simulation

According to the formula in Table 1, the weighed ammonium nitrate and water are mixed and heated to 110 °C, the measured diesel engine oil and T-154 emulsifier are heated to 90 °C. Each oil phase was poured into the dispersing machine at a speed of 1200 r min^−1^,1400 r min^−1^,1600 r min^−1^, and the aqueous solution was poured into the oil phase at a constant speed within 40 s, and the emulsion matrix was formed by stirring for 3 min. When the emulsion matrix is cooled to 55 °C, sodium nitrite and citric acid were added and mixed to obtain emulsion explosives.

**Table 1:** Formula of site mixed emulsion explosive.

Components	AN	H_2_O	Engine oil	Diesel	T-154
Mass (%)	76.2	17.3	1.79	3.09	1.62

The emulsion matrix and emulsion explosive were placed in the vacuum experimental tank, the vacuum pump was opened, the atmospheric pressure value on the vacuum experimental tank was adjusted, the vacuum experimental tank was placed in the freezer, and the temperature of the freezer was set, and the storage was 5 h. This experiment will simulate the macro and micro changes of site mixed emulsion explosives after storage in the simulated environment of 0 m, 2500 m and 4500 m altitude. The corresponding temperature and pressure at different altitudes^10,11^ are shown in Table 2.

**Table 2:** Temperature and pressure values with different altitudes.

Altitude (m)	Temperature (°C)	Pressure (Pa)
0	7	101,300
2500	− 8	74,800
4500	− 20	53,600

### Microstructure observation

Three kinds of emulsion explosives stored at different altitudes were placed on a slide, the changes of sensitized bubbles of emulsion explosives were observed under an optical microscope (Yongke Optical Instrument Co., LTD, Shanghai) with magnification of 400 times.

### Viscosity and particle size test

The viscosity trend of each emulsion matrix with temperature was measured by RVDV-1 Rotor digital viscometer (Pingxuan Scientific Instrument Co., LTD. Shanghai). The particle size of emulsion matrix was measured by Malvern MS-2000 Laser Particle Size. Test method and parameters: The emulsion matrix sample (10 g) was diluted with diesel oil (200 mL), and part of the diluent was absorbed with a disposable dropper and added to the wet sample injector of the laser particle size meter. The average diameter of the surface volume of the emulsion matrix was measured.

### Detonation velocity test

**Figure 1:**
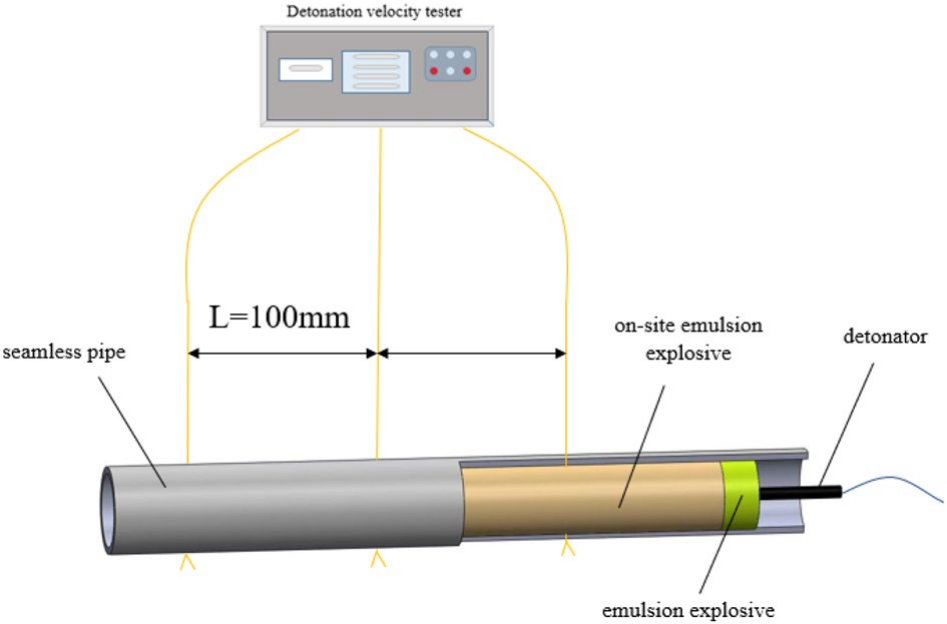
Detonation speed test device diagram.

The sample of site mixed emulsion explosive was filled into seamless steel pipe. After storage at the set temperature of the ultra-low temperature incubator, it is put into the vacuum explosion tank in time. During the experiment, the air pressure in the bunker is extracted to the corresponding value. The emulsion explosive charge was detonated by detonator, and the detonation velocity was tested by detonation velocity meter^12^. The test device is shown in Fig. 1.

### Air explosion shock wave pressure test

**Figure 2:**
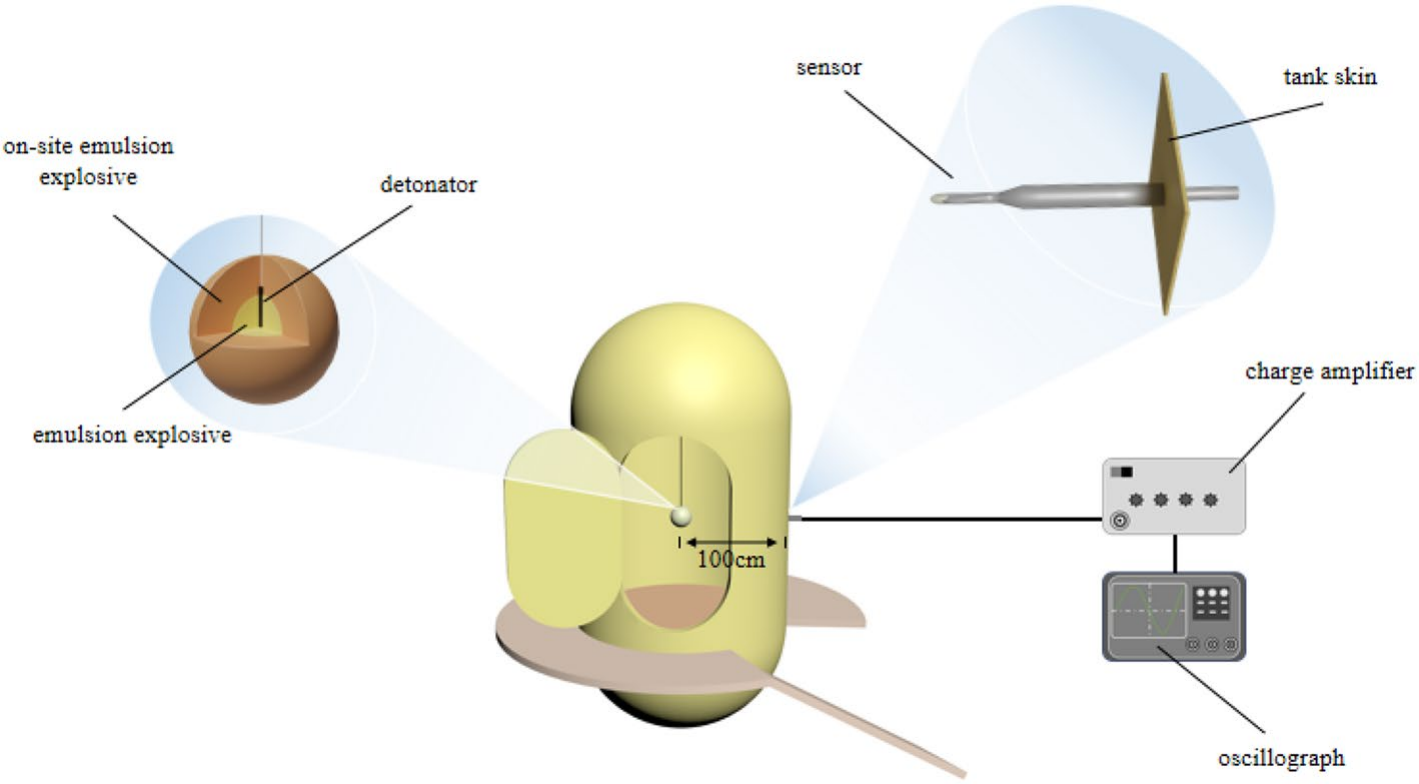
Diagram of air explosion shock wave pressure test device.

The site mixed emulsion explosive (80 g) sample and packaged emulsion explosive (20 g) as detonating charges. After storage at the set temperature of the ultra-low temperature incubator, it is put into the vacuum explosion tank in time. During the experiment, the air pressure in the bunker is extracted to the corresponding value. In the explosive bunker, fix the charge at 100 cm of the pressure sensor. The schematic diagram of the experimental setup is shown in Fig. 2.

### TG-DTG analysis

The thermal decomposition characteristics three emulsion matrix was carried out with the TGA2 (METTLER TOLEDO, Switzerland). Test methods and parameters: The emulsion matrix (2 ± 0.2 mg) were placed into alumina open crucible and heated from 20 to 400 °C at the heating rate of 5 °C min^−1^, 10 °C min^−1^, 15 °C min^−1^ and 20 °C min^−1^ respectively, and nitrogen atmosphere with the flow rate of 50 mL min^−1^ was adopted.

## Results and discussion

### Analysis of experimental results of viscosity and particle size

**Figure 3:**
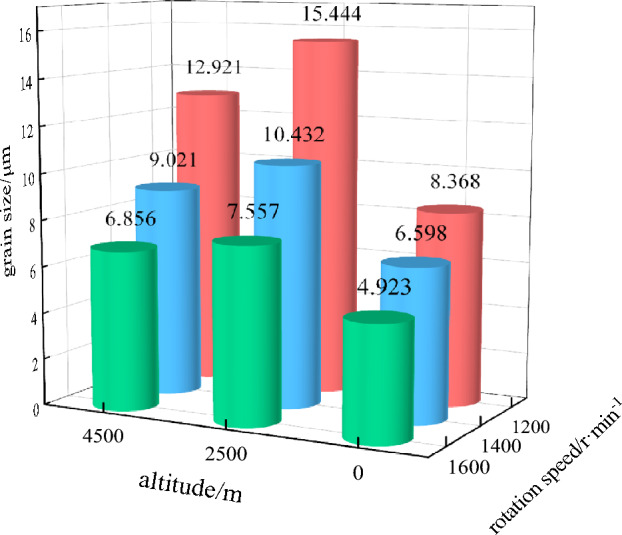
Particle size of three samples stored in different simulated environments.

The particle size of the emulsion matrix prepared at three rotational speeds after storage in three simulated environments of 0 m, 2500 m and 4500 m were shown in Fig. 3.

It can be seen from Fig. 3 that the different rotational speed of dispersion machine, and the particle size of the emulsion matrix^13^ is changed due to the different shear stress subjected to it. The samples stored in simulated environment at different altitudes was compared, it was found that the particle size of all samples first increased and then decreased with the increase of altitude. The analysis showed that after stored in a simulated environment with an altitude of 2500 m, the oil film of emulsion matrix particles gradually becomes thinner due to the action of van der waals force in a low temperature and pressure environment, and the small droplets are close to each other and finally merge into a large droplet, making the overall particle size of the system larger. At the same time, the expansion of the bubble caused by the low pressure made the droplet be squeezed and accelerated the aggregation of the droplet. After stored in a simulated environment with an altitude of 4500 m, the temperature and pressure are further reduced, and the large droplet of ammonium nitrate and inorganic salt solution in the system are the first to crystallization, resulting in the destruction of the water-in-oil structure. However, in the process of particle size test, the use of diesel as a dispersant caused that the precipitated ammonium nitrate crystals are insoluble in the dispersant, after standing, the ammonium nitrate crystal precipitates and exists at the bottom of the beaker. The solution used for measurement is the supernatant, in which there are small matrix particles.

**Figure 4:**
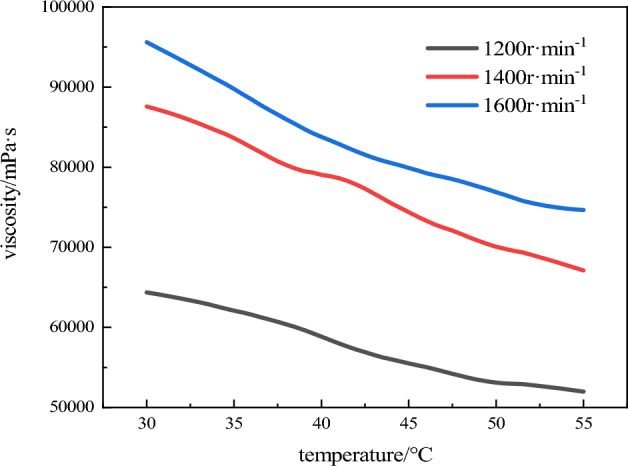
Viscosity-temperature curves of three samples.

The viscosity-temperature curves of the emulsion matrix prepared at three rotational speeds is shown in Fig. 4.

As can be seen from Fig. 4, the viscosity of the emulsion matrix prepared with three different rotational speeds increases with the decrease of temperature within a certain range. The viscosity of the emulsion matrix prepared at 1200 r min^−1^ and 1600 r min^−1^ were respectively lowest and highest. Sherman^1^ found that the emulsion matrix particles in the W/O emulsion are non-deformable spheres, and the viscosity of the W/O emulsion has the following relationship with the size of the emulsion matrix particles:

1 $$ \eta = x\frac{1}{{d_{m} }} + c $$

where: *d*_m_ is the average droplet diameter *x* and *c* are constants.

The analysis showed that there is accumulation of emulsion matrix particles in the emulsion system^14,15^, and the essence is the contact or fusion of oil films in different interface films. The oil film has fluidity, toughness, friction coefficient and hardness in the emulsion system. The smaller the particle size of emulsion matrix particles, the more the interface film contact surface, and the interaction between matrix particles in the unit volume will increase. Because the force between particles is the weak interaction, the performance is more obvious at the low shear rate, so the smaller the emulsion matrix particles, the greater the viscosity of the emulsion matrix. It can be seen by comparing Fig. 3, the emulsion matrix prepared by 1600 r min^−1^ has the smallest particle size, smallest pores between particles, and the closest contact, resulting in increased viscosity of the emulsion matrix. Therefore, the emulsion matrix prepared by 1600 r min^−1^ has the largest viscosity. The emulsion matrix prepared by 1200 r min^−1^ has the largest particle size. There is a large gap in the accumulation of emulsion matrix particles, resulting in a small amount of friction between oil films, so the viscosity of the emulsion matrix prepared by 1200 r min^−1^ is lowest. After the emulsion matrix prepared by 1200 r min^−1^,1400 r min^−1^, 1600 r min^−1^ was stored in the simulated environment from 0 to 2500 m, the particle size change rates of emulsion matrix particles were 84.56%, 58.11% and 53.50%, respectively. After being stored in simulated environment from 2500 to 4500 m, the particle size change rates of emulsion matrix particles were 16.34%, 13.53% and 9.28%, respectively. As the emulsion matrix was stored in low temperature and pressure environment, it formed large droplets through liquid drainage, which makes the overall particle size of the system increase. With the decrease of temperature and air pressure, the emulsion matrix has crystallization demulsification phenomenon, which is no longer detected by laser particle size analyzer, so the overall particle size becomes smaller. The lower the viscosity of the emulsion matrix, the smaller the binding force of the droplet, which leads to easier polymerization of droplets in the low temperature and pressure environment. The emulsion matrix prepared by 1200 r min^−1^ has large particle size and low viscosity. Under low temperature and pressure environment, droplets of emulsion matrix are more prone to polymerization, demulsification and crystallization are more likely to occur due to the low surface free energy of large particles. Therefore, the emulsion matrix prepared by 1200 r min^−1^ is better than that prepared by 1400 r min^−1^ after being stored in simulated environment at 2500 m and 4500 m above sea level. The change rate of particle size of the emulsion matrix prepared by 1600 r min^−1^ was higher.

### Microstructure test and analysis

**Figure 5:**
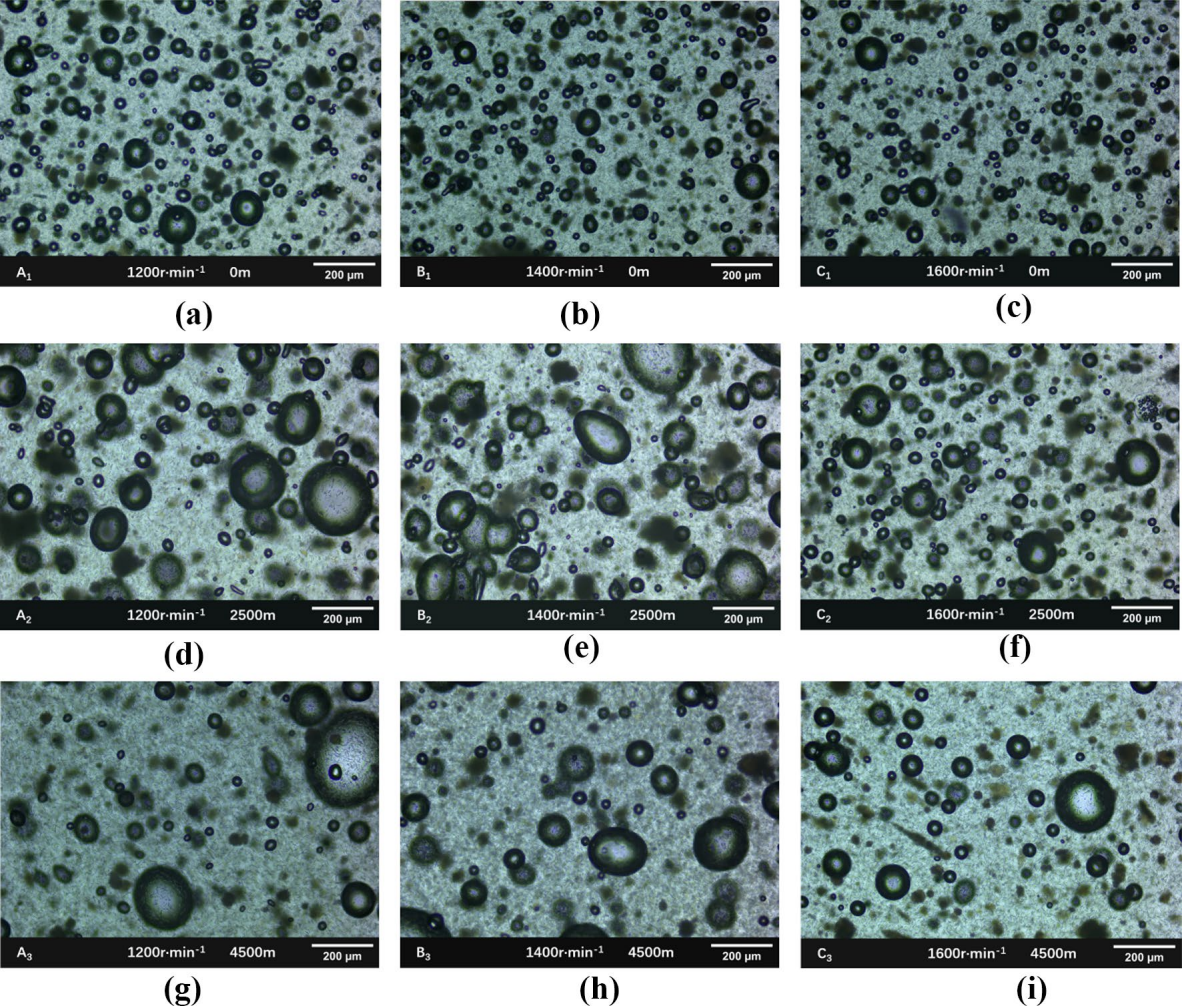
Microscopic observation of three samples stored in different simulated environments. (**a**) No. A_1_ micrograph, (**b**) No. B_1_ micrograph, (**c**) No. C_1_ micrograph, (**d**) No. A_2_ micrograph, (**e**) No. B_2_ micrograph, (**f**) No. C_2_ micrograph, (**g**) No. A_3_ micrograph, (**h**) No. B_3_ micrograph, (**i**) No. C_3_ micrograph.

The microscope was used to observe the emulsion explosives prepared at three rotational speeds and stored in simulated environments at different plateaus, and the results were shown in Fig. 5.

As shown in Fig. 5, after being stored in the simulated environment at an altitude of 0 m, the diameter of the sensitized bubbles prepared by 1200 r min^−1^ ranges from 10 to 110 μm, the number of bubbles was the largest, and the diameter of the sensitized bubble prepared by 1400 r min^−1^ ranges from 10 to 90 μm; When the shear rate was increased to 1600 r min^−1^, the diameter of the sensitized bubbles was between 10 ~ 70 μm, and the number of bubbles was smaller. It was believed that the particles of emulsion matrix prepared by 1200 r min^−1^ are smaller than the other three samples, resulting in lower viscosity and less viscous force, the bubbles were easy to expand and gather. When the sensitized bubbles were formed, the emulsion matrix with greater viscosity has a large viscous force, which hinders the expansion of bubbles, so the emulsion matrix is mostly small sensitized bubbles. However, the emulsion matrix prepared by 1600 r min^−1^ has a larger viscosity and the sensitized bubbles were not easy to expand, so the sensitized bubbles of the prepared emulsion explosive were smaller in diameter and fewer in number than other samples.

When the emulsion explosive was stored in the simulated environment of high altitude, the bubbles in the emulsion explosive prepared by three kinds of shear rate had different degrees of diffusion and accumulation and escape. The sensitized bubble^16–18^ is actually a dispersion system formed by separating the continuous phase colloid of emulsion explosive. The nitrogen generated by sensitization is the dispersion phase in this system, and the colloid is the dispersion medium. Because the large density difference between the dispersed phase and the dispersion medium, the dispersion bubbles in the colloid always had tendency to rise to the liquid surface. In the foaming process of emulsion matrix, every sensitized bubble had the same foaming power, but when the external air pressure was small, the external force on the emulsion explosive is relatively reduced, resulting in the binding force of the matrix on the sensitized bubbles becomes weakened, and the sensitized bubbles were more likely to expand and expand the volume. When two sensitized bubbles collide during the expansion process, the two bubbles would aggregate and fuse into a large bubble, and the low air pressure will accelerate this phenomenon. In addition, the site emulsion explosive matrix was a high internal ratio emulsion liquid system, which was an unstable thermodynamic state. When the preparation speed was low, the particles size of the matrix was large and the size distribution is uneven, and its structural stability was weak. After being stored at low temperature and low pressure, emulsion explosive structure would break. Nitrite ion combines with hydrogen ion produced by ionization of ammonium ion in the ammonium nitrate solution to form nitrite. However, due to the instability of nitrite, it decomposes into nitrous oxide and reacts with ammonia produced by ionization of ammonium ion to form a large amount of nitrogen, resulting in the increase in the size of sensitized bubbles.

The higher the viscosity of the emulsion with a small particle size, the stronger the ability to restrain the bubbles, and the stronger the stability of the oil-in-water structure of the emulsion explosive. When the simulated storage environment rises from an altitude of 0 m to 2500 m, the emulsion matrix prepared by 1600 r min^−1^ had higher viscosity, which better hinders the expansion and aggregation of sensitized bubbles in a low-pressure environment. Therefore, the emulsion explosive prepared by 1600 r min^−1^ had more effective bubbles after stored in a simulated environment at an altitude of 2500 m. however, the emulsion explosive prepared by 1200 r min^−1^ had a lower viscosity, the had less effective bubbles due to the large bubbles and the decrease in the number. The emulsion explosive prepared by low shear rate produced large ineffective bubbles due to the demulsification phenomenon. After being stored in a simulated environment at an altitude of 4500 m, this phenomenon is more obvious. The test results show that the emulsion matrix prepared at different shear rates has different particle size and different structural stability. Due to the interaction between emulsion matrix particles, the viscosity of the emulsion matrix was different. In the process of chemical sensitization, the larger the viscosity of the emulsion matrix, the stronger the binding ability of the sensitized bubbles, and the bubbles are not easy to expand and move during the sensitization process. Therefore, within a certain range, the higher the shear rate, the more suitable the emulsion explosive is for the plateau environment.

### Detonation speed and overpressure test analysis

The detonation velocity experiment was carried out on the emulsion explosive samples prepared by three speeds and stored in the simulated environment at different altitudes. The detonation velocity values were shown in Table 3.

**Table 3:** Detonation velocity test results.

No.	Shear rate (r min^−1^)	Altitude (m)	Detonation velocity (m s^−1^)
A_1_	1200	0	3516
A_2_		2500	3289
A_3_		4500	3108
B_1_	1400	0	3579
B_2_		2500	3457
B_3_		4500	3315
C_1_	1600	0	3684
C_2_		2500	3597
C_3_		4500	3477

As shown in Table 3, with the rise of altitude, at each speed, the detonation velocity of emulsion explosive at 4500 m decreased by 11.6%, 7.38%, and 5.62% compared to 0 m, with the shear rate increases, the detonation velocity decreases smaller. When the altitude increased from 0 to 4500 m, the emulsion explosive with a speed of 1600 r min^−1^ increased by 4.78%, 9.36%, and 11.87% compared with the speed of 1200 r min^−1^. It was believed that the viscosity of the emulsion matrix prepared at high speed is greater, it could restrain effective bubbles and improve the explosive reliability of emulsion explosive.

**Figure 6:**
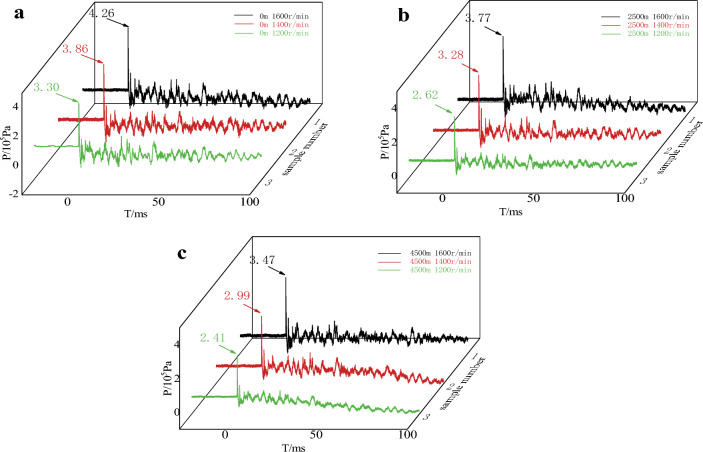
Pressure–time curve of explosive blast wave.

The pressure–time curves of the air explosion shock wave were shown in Fig. 6.

As can be seen from Fig. 6, the peak shock wave pressure of the emulsion explosive prepared by 1600 r min^−1^ was the largest under three kinds of plateau simulation environments, and the peak shock wave pressure of the emulsion explosive prepared by 1200 r min^−1^ was the smallest. At the same altitude, the peak pressure of the emulsion explosive prepared at a shear rate of 1600 r min^−1^ increased by 29.09%, 43.89%, 43.98% compared to 1200 r min^−1^, at the same shear rate, the peak pressure of the emulsion explosive of 4500 m altitude decreased 26.97%, 22.54%, 18.54% compared to 0 m.

It was analyzed that the fine emulsion particles of emulsion explosives had a larger specific surface area, so it received more energy from the thermal decomposition products, the number of activation centers were formed, which enhanced the reaction rate, shifted the decomposition temperature to a lower temperature, and the reaction interval becomes narrower, and the decomposition reaction proceeds more completely. It could be seen that, within a certain range, the increase in shear rate would increase the detonation velocity of emulsion explosive. In addition, the number of effective bubbles in the emulsion explosive would also have an impact on the detonation velocity of the samples. When the detonator detonation, the shock wave skimmed over the bubbles in the emulsion explosives so that their adiabatic compression to form a hot spot, the occurrence of the detonation reaction and rapid diffusion of the entire explosive.

After stored in the plateau environment, the bubbles inside the explosive are more likely to gather and diffuse under low temperature and pressure decreased the number of effective bubbles in the emulsion explosive. The greater the viscosity of the emulsion matrix, the stronger the binding ability of the prepared emulsion explosive to the bubbles, and the bubbles are not easy to move. Compared with Fig. 5, it can be seen that due to the large viscosity of the emulsion matrix prepared by 1600 r min^−1^, the bubbles are strongly bound to the emulsion matrix after simulated storage in a plateau environment, and the bubbles are not easy to expand and aggregate into large bubbles or escape from the emulsion explosive, forming more effective bubbles with a diameter of 1 ~ 100 μm. However, the viscosity of the emulsion matrix prepared by 1200 r min^−1^ is small, and the bubbles are more likely to shift inside the matrix, resulting in a decrease in the number of effective bubbles inside the emulsion explosive. With the increase of effective bubbles, the hot spots in the unit volume of emulsion explosive would increase and the detonation reaction will be more complete. Therefore, the smaller the internal phase of the emulsion explosive in a certain range, the better the explosive performance of the emulsion explosive, and the emulsion explosive could adapt to the plateau environment.

### TG-DTG analysis

**Figure 7:**
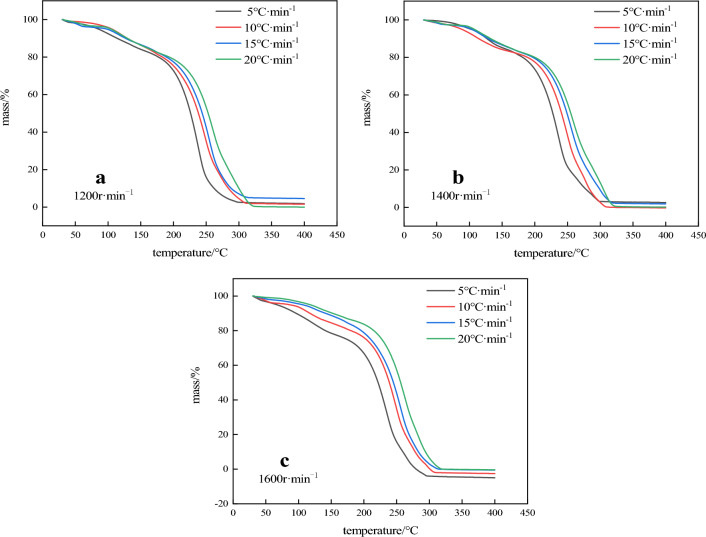
TG curves of three emulsified substrates.

**Figure 8:**
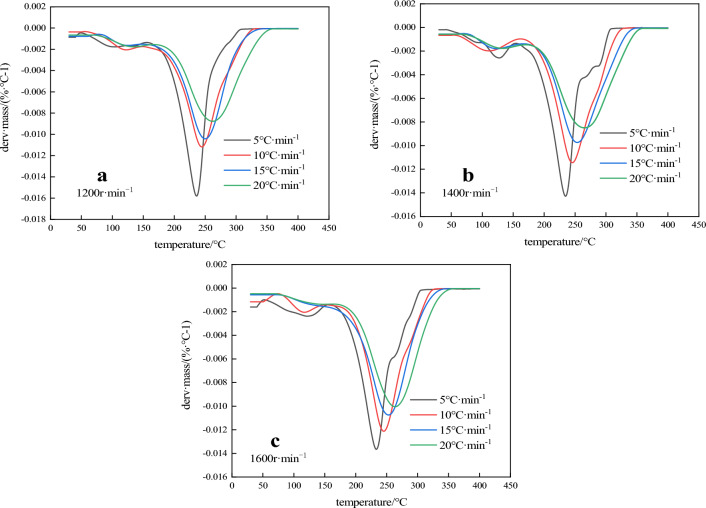
DTG curves of three emulsion matrix.

Site mixed emulsion explosive is a metastable substance with high energy. In the process of pumping and charging, external excitation will accelerate the thermal decomposition reaction, which may cause serious consequences such as explosion. In order to study the influence of thermal decomposition characteristics of site mixed emulsion substrates prepared at different rotational speeds, thermal analysis experiments were conducted on three emulsion substrates at programmed temperature. TG and DTG curves were shown in Figs. 7 and 8.

TG curves of the three samples are basically the same, indicating that their thermal decomposition processes are basically similar, and the decomposition of the emulsion matrix after heat is mainly divided into three stages. The first stage is 20 ~ 150 °C, and the mass loss rate (α) is about 16%, corresponding to an obvious mass loss rate peak on the DTG curve, indicating that the ammonium nitrate in the emulsion explosive matrix and the oil phase material do not significantly decomposition in the first stage, which is mainly caused by the heat evaporation of free water in the emulsion matrix and the separation of the inner phase. The second stage is 150 ~ 180 °C. The sample mass changes slowly and there is no obvious mass loss in this stage. For the same group of samples, the faster the heating rate, the shorter the duration of this stage, and there is no significant difference between the three groups of samples at this stage. At the third stage, when the temperature is 180 ~ 300 °C, the TG curve plummets, indicating a rapid loss of reactive substances and a violent decomposition reaction, α is about 75%, and the TG and DTG curves of each group shift to high temperature in different degrees with the increase of heating rate. As can be seen from Fig. 8, the final mass remaining was not 0% the reason why is because the sample after the completion of the test, the crucible contains residual coke after combustion, and there was an error when weighing the sample, so some curves on the image quality of about 0%. The DTG peak temperature (T_p_) of the three samples is shown in Table 4, and the DTG peak temperature (T_p_) and reaction initial temperature (T_onset_) of the samples at the heating rate of 10 °C min^−1^ are shown in Table 5.

**Table 4:** DTG peak temperature of 3 samples.

$\beta$/(K min^−1^)	*T*_p_ (°C)		
	1200 r min^−1^	1400 r min^−1^	1600 r min^−1^
5	229.72	230.03	230.87
10	244.36	245.31	245.62
15	253.13	254.01	254.83
20	263.04	263.78	264.32

**Table 5:** Initial reaction temperature and DTG peak temperature of 3 samples.

Rotation speed (r min^−1^)	*T*_onset_ (°C)	*T*_p_ (°C)
1200	199.34	244.36
1400	204.27	245.31
1600	209.36	245.62

**Figure 9:**
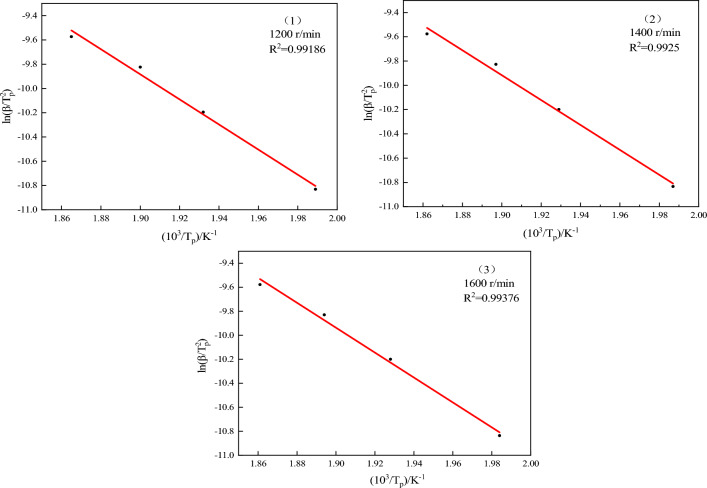
Thermal decomposition kinetics equation fitting curve.

In combination with Tables 4 and 5, it can be seen that the T_onset_ and T_p_ of the emulsion matrix prepared at three different rotational speeds increase with the decrease of the particle size of the emulsion, indicating that the particle size has great influence on the thermal decomposition performance of emulsion matrix. In order to obtain the kinetic parameters of the emulsion matrix prepared at different rotational speeds, the kinetic analysis differential method of thermal analysis curve (Kissinger method) was used to calculate the heating rate of 10 °C min^−1^ to obtain the activation energy of the reaction. The thermodynamic equation is shown in Eq. (2) ^19,20^:

2 $$ {\text{ln}}\left( {\frac{\beta }{{T_{P}^{2} }}} \right) = \ln \left( {\frac{AR}{{E_{k} }}} \right) - \frac{{E_{k} }}{{RT_{{\text{p}}} }} $$

where: β is the heating rate, K min^−1^; Tp is the peak temperature of DTG, K; A refers to the prefactor, s^−1^; E_k_ is the reaction activation energy, kJ mol^−1^; R is the universal gas constant, 8.314 J (mol K)^−1^; According to Table 4 and Eq. (2) for ${\text{ln}}\left( {\frac{\beta }{{T_{P}^{2} }}} \right) - \frac{{10^{3} }}{{T_{{\text{p}}} }}$,the DTG peak temperature at each heating rate was linearly fitted, and the fitting results of the three samples were shown in Fig. 9. R^2^ was greater than 0.99 indicated having a high linear fit. The reaction activation energy (E_k_) of each group was calculated from the slope of the fitted line. The calculated results of emulsion matrix samples prepared at the speed of 1200 r min^−1^, 1400 r min^−1^ and 1600 r min^−1^ were 80.47 kJ mol^−1^, 85.32 kJ mol^−1^ and 91.04 kJ mol^−1^, respectively. It can be seen that the thermal decomposition activation energy of the site mixed emulsion matrix increases with the decrease of the inner phase particle size, and the emulsion matrix prepared by 1600 r min^−1^ is 13.14% higher than that prepared by 1200 r min^−1^. The analysis shows that when the inner phase particle size of the emulsion matrix is small, the uniformity and stability of the particles are greater. The reduction of the oil film thickness improves the consistency of the system, thus slowing down the heat accumulation rate inside the matrix, and thus improving the thermal decomposition activation energy inside the emulsion matrix.

## Conclusion

In conclusion, site mixed emulsion explosive with different particle sizes can be prepared by varying the shear rate, and the viscosity increases with the increase of the internal phase particle size. Under the same foaming conditions, the number and size of sensitized bubbles decreased with the decrease of internal phase particle size. After storage in a plateau environment, the internal phase particle size increased and then decreased, and the number of effective bubbles decreases with elevation. The detonation performance of the emulsion explosives decreased when the altitude was increased, and the emulsion explosives prepared at high shear rate could effectively improve the detonation performance of the explosive. The activation energy of thermal decomposition of the emulsion matrix increases with the decrease of particle size. Increasing the shear rate to reduce the internal phase particle size was conducive to the application of emulsion explosive in the plateau environment. After micro and macro performance test, it as found that the stability and detonation performance of site mixed emulsion explosive with small inner phase particle size would have better stability and detonation performance at high altitudes.

### Supplementary Information

Supplementary Information.

## Data Availability

All data generated or analysed during this study are included in this published article and its supplementary information files.
